# Lithocholic Acid Species: Metabolism, Signaling Pathways, and Clinical Significance in Enterohepatic Diseases

**DOI:** 10.3390/ijms262311530

**Published:** 2025-11-28

**Authors:** Lianggui Leng, Guangzeng Zhou, Ana Liu, Huiying Wang, Yan Ni

**Affiliations:** 1 School of Pharmacy, Shandong University of Traditional Chinese Medicine, Jinan 250355, China; dxjandlx@163.com (L.L.); 15653853370@163.com (G.Z.); 2 Children’s Hospital, Zhejiang University School of Medicine, National Clinical Research Center for Child Health, Hangzhou 310052, China; lwtgyx@outlook.com (A.L.); wanghuiyingny@163.com (H.W.)

**Keywords:** lithocholic acid species, enterohepatic diseases, bile acid metabolism, microbial transformation, receptors

## Abstract

Secondary bile acids are generated from the metabolism of primary bile acids by intestinal flora and play important roles in lipid digestion, regulation of metabolic homeostasis, and intestinal-hepatic axis signaling. Recent studies indicate that lithocholic acid (LCA) and its derivatives (e.g., 3-oxoLCA and isoLCA) are significantly dysregulated in inflammatory bowel disease, nonalcoholic fatty liver disease, and hepatocellular carcinoma. Consequently, LCA species are emerging as promising biomarkers and potential targets for early diagnosis. This review systematically summarizes the metabolic pathways of LCA species, their distribution and concentrations in human blood, urine, and fecal samples, as well as the progress of recent research studies on enterohepatic disorders, which will serve as a reference for the development of new diagnostic and therapeutic methods in the future.

## 1. Introduction

Bile acids (BAs) are synthesized from cholesterol in hepatocytes and regulate glucose, lipid, steroid and xenobiotic metabolic homeostasis in humans and animals [1]. As signalling molecules, bile acids exhibit metabolic dysregulation under pathological conditions, rendering them potential biomarkers for diagnosing various diseases. Recent studies have integrated biochemical indicators with serum bile acid biomarkers to construct disease prediction models, offering non-invasive detection methods that facilitate early disease detection and intervention [2,3]. Bile acids represent a focal point in metabolomics research. Current studies, through the refinement of mass spectrometry techniques, continue to identify novel bile acids. This is complemented by the application of artificial intelligence to assist in the identification of bile acid-related metabolic enzymes, thereby advancing developments within the bile acid field [4]. LCA is a secondary bile acid synthesised via an alternative pathway. It is formed through the 7α-dehydroxylation of chenodeoxycholic acid (CDCA) under the influence of the gut microbiota and has garnered attention due to its role in intestinal and hepatic diseases [5]. Advances in microbiome and metabolome technologies have revealed LCA and its novel derivatives in a variety of diseases, especially those related to the enterohepatic circulatory system, which are closely related to BA synthesis and metabolism. Further studies have demonstrated the immunomodulatory functions of novel LCA derivatives, including 3-oxoLCA, iso-LCA, and isoallo-LCA, in inflammatory bowel disease (IBD) and metabolic dysfunction-associated steatotic liver disease (MASLD) [6,7,8]. Recent studies also report anti-tumor, skeletal muscle regenerative, and anti-aging properties of LCA [9,10,11]. However, LCA may exacerbate skin inflammation [12] or steatohepatitis through distinct mechanisms. Given these multifaceted roles, LCA species are increasingly recognized as critical regulators of human health and diseases. This review comprehensively examines the metabolic pathways, tissue distribution, and clinical relevance of LCA species, providing a foundation for future diagnostic and therapeutic innovations.

## 2. Metabolic Pathway and Microbial Transformation of LCA Species

**Figure 1:**
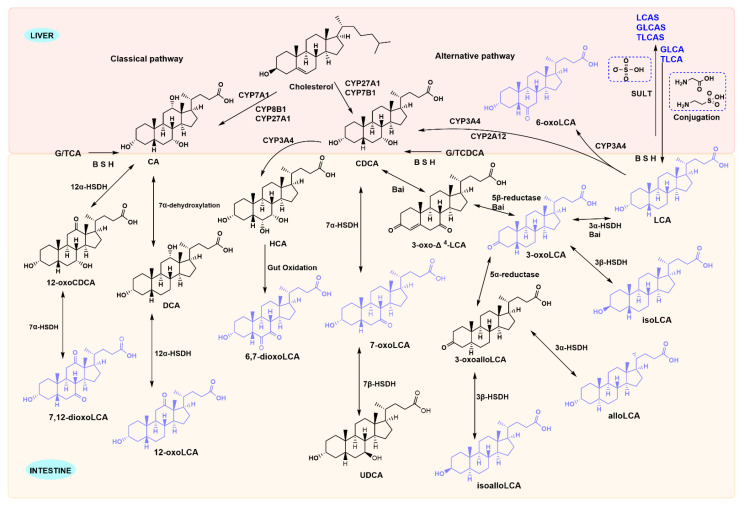
Metabolic pathways of LCA species. Primary bile acids generated in the liver are converted into LCA species by gut microbiota and host enzymes. However, 6-oxo-LCA is formed when LCA is reabsorbed into the liver via enterohepatic circulation and subsequently metabolised by CYP3A4 enzymes. LCA reabsorbed into the liver conjugates with glycine and taurine to form TLCA and GLCA, respectively. All three undergo sulphonation metabolism in the liver via the action of hepatic sulphotransferase enzymes.

Advances in analytical techniques have expanded the characterization of LCA derivatives. We summarize their interconversion pathways and key metabolic enzymes in Figure 1. In hepatocytes, cholesterol is converted to primary BAs by the enzymes cholesterol 7α-hydroxylase (CYP7A1) and sterol 27 hydroxylase (CYP27A1). These Primary BAs are then conjugated to taurine or glycine in the liver, stored in the gallbladder and released into the duodenum during food intake. There they undergo microbial transformations in the intestine [13]. Conjugated BAs undergo deconjugation via intestinal bacterial bile salt hydrolases (BSH) enzymes, subsequently being converted into secondary BAs by 7α-dehydroxylation [14]. Both conjugated and unconjugated LCA are actively transported with high affinity by intestinal BA transporter protein (IBAT) from the terminal ileum into the enterocytes. There, they bind to Fatty Acid Binding Protein 6 (FABP6) and are transported via organic solute transporter α (OSTα) and organic solute transporter β (OSTβ), expressed on the intestinal cell basement membrane, ultimately return to the liver via the portal venous circulation [15]. LCA is present in the human body at low concentrations, but it can be toxic to the liver if present in high concentrations. Around five percent of bile acids that are not absorbed will enter the large intestine and are excreted in faeces, while a small quantity of LCA that returning to the liver is secreted into bile after sulphation, then excreted in urine via the kidneys through the bloodstream [16].

In addition, LCA species are mainly generated through four pathways under the action of gut microbiota: deconjugation (removal of glycine or taurine), 7α-dehydroxylation, oxidation, and isomerization [13]. BSH enzymes can hydrolyze conjugated bile acids that are bound to glycine and taurine, while hydroxysteroid dehydrogenase is responsible for the biotransformation of primary BAs into secondary BAs. CYP3A4 is the enzyme involved in the biotransformation of LCA in liver microsomes to form metabolites with LCA as the parent structure (such as 3-keto-LCA and 6-keto-LCA). The formation of these LCA derivatives enhances hepatic detoxification and elimination functions [17]. Enzymes involved in LCA metabolism are gradually being characterized in microorganisms (Table 1). BSH enzyme activity have been expressed in gut microbiota, including *Clostridium*, *Bacteroides*, *Lactobacillus*, *Bifidobacterium*, *Enterococcus*, and *methanogenic bacteria*. Gut microbiota members with 3α-hydroxysteroid dehydrogenase (3α-HSDH), bile acid 5β-reductase, and bile acid 5α-reductase produce allo-secondary bile acids via 3-oxo-∆^4^-LCA intermediate that resets the ring stereochemistry [18]. 7α-HSDH has been primarily characterized in human gut microbiota species such as *C. absonum, C. hiranonis, and C. hylemonae*. Phylogenetic analysis indicates that 12α-HSDH is widely distributed across the *Actinobacteria* phylum, the *Coriobacteriaceae* family, and human gut *archaea* [19]. Recent studies have proposed a fifth microbial modification, recombination, which includes the formation of BA-24-amides with amino acids or polyamines at the C-24 position and BA-3-O-acyl esters with fatty acids or organic acids at the C-3 position [20]. Research has shown that BAs decorated by the gut microbiota do not only bind only to glycine, taurine at the C-24 position, but also produce more than 200 amino acid-BA conjugates [20,21,22]. Sato et al. found a series of LCA derivatives produced by enriched gut microorganisms in feces of Japanese centenarians, including 3-iso-, 3-oxo-, 3-allo-, 3-isoallo-, and 3-oxoallo-LCA [18].

## 3. LCA-Receptor Signaling Pathways in Maintaining Metabolic Homeostasis

BAs function as signaling molecules that attach to specific receptors, such as the farnesoid X receptor (FXR), G protein-coupled receptor 5 (TGR5), vitamin D receptor (VDR), pregnane X receptor (PXR), liver X receptor (LXR) and constitutive androstane receptor (CAR). Previous studies have summarized the roles of LCA in anti-inflammation, immune regulation, and glycolipid metabolism modulation, which are mediated through the activation of signaling pathways involving the receptors mentioned above [26]. The potency of unconjugated BAs in activating FXR is as follows: CDCA > DCA > LCA ≫ CA, while the most effective endogenous ligands for TGR5 are LCA and its derivatives [27,28,29]. Upon activation by BAs, the intestinal FXR regulates BA levels within the enterohepatic circulation. TGR5 mainly participates in the regulation of glucose, lipid, and energy metabolism after BAs activation [5,30]. Beyond acetylated DCA and CA, LCA and its oxidative metabolite 3-oxo-LCA are also primary bile acid ligands for PXR, whereas conjugated bile acids fail to activate PXR [31,32]. VDR exhibits heightened sensitivity to bile acids compared to FXR and PXR, demonstrating particularly pronounced responses to ketone LCAs and glycine-LCA within the LCA species [33]. The ketone derivatives of LCA, 6-keto and 7-keto-LCA, have been suggested to exert a trans-inhibitory effect on CAR activity [32,34]. Up-regulation of PXR expression, on the one hand, inhibits BA synthesis by blocking transcriptional activation of the promoter region of the CYP7A1 gene via the SHP-LRH-1 pathway and suppressing its gene expression. On the other hand, PXR serves as key transcription factor to govern the inducible expression of the CYP3A gene, accelerating the metabolism of drugs in vivo and potentially reducing the efficacy of these drugs or generating adverse effects [35]. It has been shown that PXR has the capacity to counteract the adverse impacts of toxic hydrophobic bile acids (e.g., LCA) by activating cytochromes. These enzymes hydroxylate BAs to less toxic and more hydrophilic BA species. Furthermore, PXR induces sulphatase activity, thereby facilitating the second phase of BA metabolism and the detoxification process [36]. The regulatory mechanisms governing the metabolic cascade of the vitamin D receptor (VDR) bear a strong resemblance to those mediated by other nuclear receptors. These receptors function as lipid sensors to mediate the detoxification of their ligands. LCA may activate feedforward catabolic pathways by binding to VDR, thereby increasing CYP3A expression and ultimately achieving LCA detoxification [37]. Concurrently, inducing sulfotransferase expression leads to LCA sulphation, which inhibits its passive uptake by enteric pericytes and promotes its excretion [38].

## 4. Distribution and Concentrations of LCA Species in Human Biospecimen

Next, we systematically summarized the distribution and concentrations of LCA species in the blood, urine, and fecal samples of healthy subjects that have been reported in previous studies. First, more studies prefer the identification of BAs in the blood samples than urine or fecal samples, and there are differences in the distribution and concentration of LCA species in different sample types. We noticed that most of the LCA species can be detected in blood samples, mostly glycine-conjugating LCA analogs. Interestingly, LCA concentrations increase during infancy and early childhood (0–3 years) but stabilise with advancing age [39] (Table 2). This phenomenon confirms that intestinal bile acid metabolism undergoes phased maturation alongside microbiota development, by which stage the microbiota has matured into an adult-type composition [40]. In addition, the potential biological significance of the evolution of LCA levels in the human body at different ages for growth and development remains to be further studied. In urine samples, the LCA series are dominated by the II phase metabolite 3-sulfated-LCA in high concentrations (Table 3). Among the secondary bile acid LCA derivatives produced by the conversion of primary bile acids by gut microbiota, LCA remains the most abundant in fecal samples (Table 4). Due to the difficulty in obtaining liver samples, only one study has reported the concentration of LCA in normal liver tissue. This research selected liver parenchymal tissue from the perilesional area of patients with focal liver disease (*n* = 6, aged 40–60 years) as samples. Using gas chromatography-mass spectrometry, the LCA concentration was detected as 1.5 ± 0.2 nmol/g (mean ± SEM) [41].

## 5. Clinical Significance of LCA Species in Enterohepatic Diseases

### 5.1. Diagnostic Biomarkers

Numerous studies have shown that MASLD patients have increased levels of total BAs, increased hepatic BA synthesis, and intestinal flora disorders leading to an imbalance in secondary BA metabolism, which promotes disease progression [52]. Higher levels of BAs in both serum and feces have been strongly associated with the severity of liver fibrosis in NAFLD patients [53,54]. LCA, GLCA, and TLCA were significantly elevated in both liver fibrosis patients and in three distinct C57BL/6J mouse models of liver fibrosis induced by high-fat diet (HFD), streptozotocin-high-fat diet (STZ-HFD) and carbon tetrachloride (CCl_4_), respectively. Notably, the *Pseudomonas* species significantly increased in the HFD-induced model showed a significant positive correlation with LCA and GLCA [55]. In our large cohort study using biopsy-confirmed NAFLD, patients with mild fibrosis (F1) had a significant increase in secondary BAs, including DehydroLCA, 6-ketoLCA, and TLCA. These were associated with clinical indicators to construct and validate a liver fibrosis prediction model. In the early stage (F1 vs. F0) non-obese patient cohort (*n* = 119), the training set AUROC value was 0.78 and the testing set AUROC value was 0.69. This outperformed the traditional non-invasive Hepamet fibrosis score, offering a novel strategy for non-invasive detection of early-stage liver fibrosis [3]. The above studies demonstrate the LCA species could serve as early MASLD markers and key measures of disease advancement.

Furthermore, when examining the changes in BA profiles during NAFLD progression, plasma 7-ketoLCA levels were found to be significantly elevated in patients with advanced steatosis and were associated with the progression of NASH, hepatocellular edema, and steatosis [56]. Interestingly, the BA profile characteristics of NAFLD patients at risk for gallstone disease exhibit gender specificity. Compared to males, females demonstrate significantly elevated serum concentrations of GLCA, isoLCA, and 12-ketoLCA, alongside markedly increased fecal LCA levels. Conversely, fecal 7-ketoLCA levels are significantly reduced [57]. In patients with cirrhosis with sarcopenia, serum secondary BAs (total DCA, total LCA, unconjugated DCA, and unconjugated LCA) and fecal total LCA were markedly higher than in cirrhotic patients without sarcopenia [58]. Recent research has indicated that microbiota-generated metabolites contribute to hepatocellular carcinoma (HCC) development, and BAs may serve as biomarkers of HCC risk [59]. Serum iso-LCA levels and fecal abundance of *B. ovatus* were significantly increased in patients with HCC, both of which may serve as biomarkers for HCC diagnosis [60].

### 5.2. Therapeutic Targets

#### 5.2.1. Direct Effects of LCA Species on Intestinal Diseases

In vitro cellular experiments demonstrated that LCA inhibited deoxynivalenol (DON)-induced intestinal inflammatory responses and oxidative stress in intestinal epithelial cells (IPI-2I) by modulating PPARγ-mediated core inflammatory and antioxidant genes [61]. Furthermore, LCA reverses DON-induced apoptosis in IPI-2I cells by reducing the expression of cleaved caspase-3 and PARP-1. Further investigations revealed that LCA reduces elevated bile acid levels in IPI-2I caused by DON-promoted expression of the rate-limiting bile acid synthase CYP7A1. It significantly downregulates CYP7A1, CYP7B1, CYP8B1, and CYP27A1 expression, a regulatory process likely closely associated with the nuclear receptor RORγ [62]. In vivo animal experiments verified that LCA treatment significantly reduced a variety of chemokines and cytokines associated with intestinal inflammation, such as chemokine ligands CCL5, CXCL10, IL-17A and TNF-α [63]. 7-Keto-LCA was shown to be an FXR antagonist that promotes Wnt signaling in an aspirin-induced C57BL/6J mouse model of intestinal injury, thereby facilitating self-renewal of intestinal stem cells [64]. 12-ketoLCA inhibits the secretion of pro-inflammatory cytokine IL-17A by promoting the expression of the nuclear receptor VDR in ILC3s, which may be important for its efficacy against colitis [65]. Similarly, isoalloLCA attenuates intestinal inflammation by increasing the binding of the orphan nuclear receptor NR4A1 at the Foxp3 locus and enhancing the transcription of the Foxp3 gene to induce the differentiation of naïve T cells into Treg cells [8]. The above studies suggest that LCA species play a beneficial role in the gut, especially through immune modulation. However, when the intestinal barrier is damaged, tight junctions break down, allowing more LCA species to diffuse from this site to various tissues and organs throughout the body. When the concentration reaches a certain level, it might trigger various inflammatory responses.

#### 5.2.2. Direct Effects of LCA Species on Hepatic Diseases

**Figure 2:**
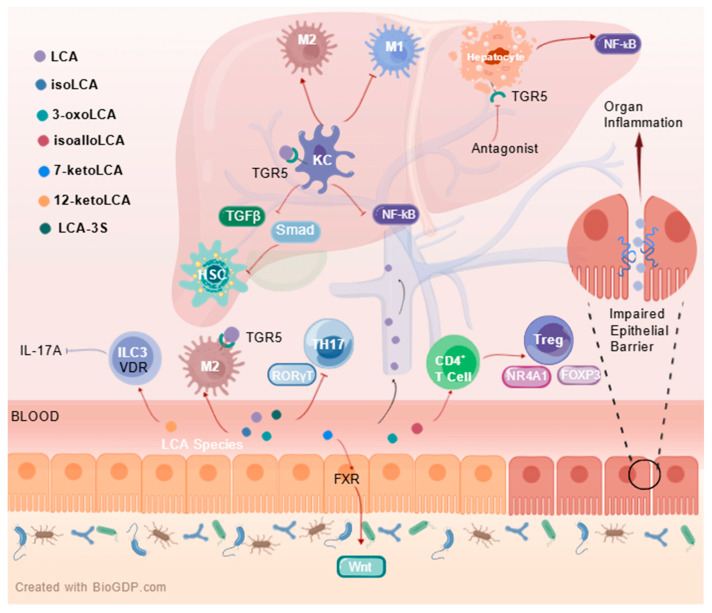
Immune signalling pathways and mechanisms of LCA species in the enterohepatic axis. Created with http://BioGDP.com [73].

The enterohepatic axis pathway is increasingly recognized internationally, and the role of LCA in the gut can further influence liver function. It was noted that LCA downregulates the expression of proteins critical to the NF-κB inflammatory signaling pathway, such as IκBα and NF-κB p50 phosphorylation, and nuclear translocation of NF-κB p65. It exerts anti-inflammatory and hepatoprotective effects on Klebsiella pneumoniae-infected mice via TGR5 [66]. LCA has been demonstrated to inhibit the activation of HSCs by reducing the activation of transforming growth factor β (TGF-β) Smad-dependent and Smad-independent pathways and inducing apoptosis. Meanwhile, LCA suppresses glycolysis and enhances oxidative phosphorylation, driving hepatic macrophage polarization toward M2 phenotype while preventing their transformation into M1 type [67]. In macrophages, the NF-κB pathway activation is primarily linked to the cell’s capacity to enhance inflammation. TGR5 reduces inflammation in liver macrophages by suppressing the activation of the NF-κB pathway, which is in contrast to its role in hepatocytes [68]. Another study reported that LCA, 3-oxoLCA and isoLCA depend on and up-regulate TGR5 to promote M2 macrophage polarization and inhibit TH17 cell differentiation, but also directly inhibit the expression of RORγt, which drives the differentiation of TH17 cells, to exert anti-inflammatory effects [69]. The reduction of LCA-3-S due to BA metabolism disorders may be one of the pathogenic mechanisms of cholestatic liver disease. It has been suggested that LCA-3-S binds better to RORγt than 3-oxo-LCA. It specifically inhibits TH17 cell differentiation by targeting RORγt without affecting TH1, TH2 and Treg cells [70]. Recently, it was found that isoLCA administration to HFD-induced C57BL/6J mice NASH model prevented the progression of NAFLD to NASH by regulating the expression of genes related to TH17 cell differentiation/IL-17/PPAR signaling pathway to inhibit TH17 cell differentiation and promote Treg cell proliferation [71]. Administration of alloLCA in the HFD and CCl_4_-induced C57BL/6J mouse model of MASH corrected the disruption of various pathways linked to immune, inflammatory, and metabolic signaling, indicating that alloLCA might play a role in treating chronic inflammation and fibrosis-associated liver diseases (Figure 2) [72].

Our studies have reported that conjugated LCA activates hepatic TGR5 to promote lipotoxicity and the transition of MASLD to MASH by disrupting carnosine biosynthesis [74]. Notably, LCA plays a negative role in treating the progression of hepatocellular carcinoma. AKR1D1, an enzyme involved in BA metabolism, plays an important tumor suppressor role in hepatocellular carcinoma, and its deficiency significantly accelerated the progression of hepatocellular carcinoma by a mechanism that may be a significant increase in secondary BA iso-LCA impairs the cytotoxic function of CREB1 by inhibiting its phosphorylation in NK cells, dose-dependently decreasing the ability of NK cells to kill tumor cells [60]. LCA, a secondary BA accumulated in the microenvironment of hepatocellular carcinoma, can promote tumorigenesis and T cell dysfunction in vivo by inducing endoplasmic reticulum (ER) stress in tumor-specific T cells [75]. In summary, the application of LCA and other derivatives in the field of liver disease needs to be further explored.

#### 5.2.3. Indirect Effects of LCA Species on Enterohepatic Diseases

In addition to direct intervention with LCA species, many studies have attempted to target BA receptors, BA synthases, and gut flora to regulate secondary BA metabolic homeostasis in vivo. Secondary BA synthesis is regulated by remodeling the gut microbiota including *Parabacteroides*, *Clostridium*, and *Akkermansia muciniphila*, which encode 7α- HSDH [76,77,78]. FXR and TGR5 are two of the most extensively researched BA receptors. The agonists that have been developed for these related receptors, which inhibit NF-κB pathway-mediated pro-inflammatory cytokine releases, have demonstrated their efficacy in ameliorating hepatic steatosis and inflammation in the clinic [79,80]. The activation of the NF-κB pathway in hepatocytes is generally seen as protective, particularly during inflammatory damage, as it enhances the expression of genes that prevent apoptosis and supports liver function [74]. The TGR5 receptor, which is highly expressed on hepatocytes following disease onset, inhibits NF-κB signalling when activated by LCA, thereby exacerbating disease progression. This suggests that specific inhibitors targeting hepatocyte TGR5 may represent a viable therapeutic strategy. Atorvastatin increases BA synthesis and regulates the gut microbiota by promoting the abundance of 7-dehydroxylase-expressing *Clostridium*, increasing LCA level and activating TGR5, and attenuating liver injury in a diet-induced steatohepatitis model [77]. By binding to PXR and VDR, LCA derivatives increase the excretion of cytotoxic LCA and prevents cholestatic liver injury and steatohepatitis due to liver accumulation [81]. Gly-β-MCA prevents cholestatic liver injury by promoting fecal BAs excretion and reducing colonic LCA exposure [82].

## 6. Conclusions

A total of 15 LCA species have been quantitatively identified and reported in the blood, urine and fecal samples. Yet, more novel compounds are to be unearthed with the development of mass spectrometry. LCA derivatives have been reported to play roles in anti-inflammatory, reducing lipid accumulation, antiviral and antibacterial activities. Although LCA and its derivatives have shown promising therapeutic targets in the field of enterohepatic diseases, the dual effects of LCA (protection and toxicity) that dependent on the concentrations, tissue microenvironment and species differences in bile acid metabolism, and the precise regulatory mechanisms have not been fully elucidated. Thus, more systematic work is required to explore the specific roles of LCA and its derivatives in the gut and liver. Furthermore, existing research is largely confined to cellular or animal models, necessitating further validation to determine whether the mechanisms observed in animals are applicable to humans. Individual differences in gut microbiota composition may significantly influence LCA metabolism and therapeutic efficacy, and future research should integrate multi-omics technologies to explore personalised intervention strategies; The dynamic changes of LCA across different disease stages (e.g., progression from MASLD to MASH) and its therapeutic window require systematic investigation.

In summary, how LCA interacts with the gut microbiota to exert its effects in the gut and avoid enterohepatic circulation-induced damage to other organs warrants further investigation. Future efforts should also focus on technological advancements, the discovery of new compounds, and in-depth mechanistic studies of the association between LCA metabolic networks and disease, with the aim of developing multi-target therapies based on the microbiota-BA axis. This approach holds the promise of providing novel diagnostic tools and treatment strategies for enterohepatic diseases.

## Figures and Tables

**Table 1:** Bacterial taxa involved in the metabolism of LCA species.

Enzymes	Bacterial Taxa	Substrate	Product	Refs.
BSH	*Clostridia* *Bacteroides* *Enterococcus* *Bifidobacteria* *Lactobacilli* *methanogenic archaea*	Conjugated bile acids or free bile acids	Unconjugated bile acids	[15]
5α-reductase	*P. goldsteinii* *B. theta.* *A. onderdonkii*	3-oxo-∆^4^-LCA	3-oxoalloLCA	[18]
5β-reductase	*H. hathewayi* *Lachnospiraceae*	3-oxo-∆^4^-LCA	3-oxoLCA	[18]
3α-HSDH	*R. timonensis* *Lachnospiraceae* *P. distasonis*	LCA 3-oxoalloLCA	3-oxoLCA alloLCA	[18]
3β-HSDH	*O. laneus* *Odoribacteraceae* *P. distasonis* *P. merdae*	3-oxoLCA 3-oxoalloLCA	isoLCA isoalloLCA	[18]
7α-HSDH	*C. absonum* *C. hiranonis* *C. hylemonae* *Eubacterium* sp.	CDCA	7-oxoLCA	[19,23,24]
12α-HSDH	*C. scindens* *C. hiranonis* *C. hylemonae* *Eggerthella* sp.	12-oxoLCA	DCA	[19,25]

**Table 2:** Concentrations of LCA species in the blood samples of healthy individuals.

Sample Type	Plasma	Plasma	Serum	Serum	Serum	Serum
Age	/	2 months	6~24 months	19~65 years	54.5~71.3 years	21~31 years
Instrument type	UPLC–MS/MS	HPLC	UPLC–HRMS	LC–MS/MS	LC–MS/MS	UPLC–TQMS
sample size	*n* = 43 Mean ± SD	*n* = 32 Median (IQR)	*n* = 15 Mean ± SD	*n* = 90 Mean ± SEM	*n* = 1670 Median (IQR)	*n* = 50 Mean ± SEM
LCA	3.98 ± 4.07	/	10 ± 20	7.50 ± 0.38	28.2 (19. 5–39.2)	20.06 ± 13.97
isoLCA	1.48 ± 2.73	/	/	4.93 ± 0.28	16.9 (8. 8–30.3)	37.22 ± 39.41
alloLCA	/	/	/	/	/	4.46 ± 4.20
isoalloLCA	1.08 ± 0.71	/	/	/	/	/
GLCA	14.42 ± 27.68	300 (200–480)	40 ± 60	10.7 ± 0.8	7.9 (4.2–17.3)	11.15 ± 8.34
TLCA	1.21 ± 1.99	250 (130–400)	80 ± 90	1.64 ± 0.15	4.0 (3.0–6.1)	2.05 ± 1.82
3-oxoLCA	0.8 ± 1.31	/	/	/	1.4 (0.1–3.3)	/
6-oxoLCA	/	/	/	/	0.7 (0.1–1.5)	/
7-oxoLCA	14.61 ± 23.82	/	/	5.90 ± 0.57	12.7 (8.2–22.4)	7.92 ± 12.54
12-oxoLCA	2.36 ± 4.02	/	/	8.58 ± 0.97	4.4 (1.7–9.6)	14.44 ± 11.4
6,7-dioxoLCA	3.06 ± 5.55	/	/	/	/	ND
7,12-dioxoLCA	7.12 ± 9.05	/	/	/	/	/
LCA-3-S	/	/	/	5.36 ± 0.54	7.5 (3.4–15.3)	20.87 ± 28.21
GLCA-3-S	130.94 ± 261.36	/	/	275.5 ± 15.0	/	509.08 ± 468.34
TLCA-3-S	0.68 ± 1.28	/	/	83.0 ± 5.1	/	90.32 ± 84.24
Refs.	[42]	[43]	[39]	[44]	[45]	[46]

Note: The concentration is expressed in nmol/L, with unit conversions provided in references [39,43].

**Table 3:** Concentrations of LCA species in the urine samples of healthy individuals.

Sample Type	Random Urine	Random Urine	Random Urine	24 h Urine	Random Urine
Age	19~65 years	21~31 years	/	2.98~18.37 years	22~23 years
Instrument type	LC–MS/MS	UPLC–TQMS	HPLC–MS/MS	LC–MS/MS	LC/ESI–MS/MS
sample size	*n* = 90 Mean ± SEM	*n* = 50 Mean ± SEM [nmol/mmol creatinine]	*n* = 39 Mean ± SD	*n* = 80 Mean ± SD	*n* = 12 Mean ± SD [nmol/mmol creatinine]
LCA	0.15 ± 0.02	0.28 ± 0.26	ND	ND	/
isoLCA	ND	ND	/	/	/
alloLCA	/	ND	/	/	/
isoalloLCA	/	/	/	/	/
GLCA	0.36 ± 0.03	ND	ND	ND	/
TLCA	0.32 ± 0.03	ND	ND	ND	/
3-oxoLCA	/	ND	/	/	/
6-oxoLCA	/	ND	/	/	/
7-oxoLCA	2.27 ± 0.55	0.16 ± 0.26	/	/	/
12-oxoLCA	2.17 ± 0.48	0.18 ± 0.34	/	/	/
6,7-dioxoLCA	/	0.03 ± 0.14	/	/	/
7,12-dioxoLCA	/	0.1 ± 0.23	/	/	/
LCA-3-S	7.19 ± 0.63	2.76 ± 5.36	ND	4. 2 ± 13.5	ND
GLCA-3-S	8082 ± 46.1	109.6 ± 110.79	185.73 ± 151.63	580.6 ± 637.6	73.99 ± 68.71
TLCA-3-S	222.9 ± 12.5	17.96 ± 19.44	108.6 ± 95.39	234.3 ± 232.5	17.10 ± 18.06
Refs.	[44]	[46]	[47]	[48]	[49]

Note: Except for the concentrations in References [46,49], which are creatinine-corrected, the unit of concentration for the others is nmol/L.

**Table 4:** Concentration of LCA species in the fecal samples of healthy individuals.

Sample Type	Freeze-Dried Fecal Sample	Freeze-Dried Fecal Sample	Freeze-Dried Fecal Sample	Fresh Fecal Sample
Age	21~31 years	/	23~78 years	≥18 years
Instrument type	UPLC–TQMS	UPLC/MRM–MS	LC–MS/MS	LC–MS/MS
sample size	*n* = 50 Mean ± SEM	*n* = 43 Mean ± SD	*n* = 17 Median (Min, Max)	*n* = 136 Median (IQR)
LCA	3643.61 ± 2385.74	1016.60 ± 647.31	8401.19 (2112.54, 23,499.31)	549.79 (375.44–764.44)
isoLCA	480.8 ± 455.9	/	/	/
alloLCA	37.6 ± 62.36	/	/	/
isoalloLCA	/	/	/	/
GLCA	0.64 ± 0.73	6.68 ± 18.49	5.06 (0.00, 8.59)	0.13 (0.08–0.23)
TLCA	2.4 ± 2.05	0.51 ± 0.40	0.32 (0.00, 8.90)	0.27 (0.10–0.75)
3-oxoLCA	402.77 ± 600.14	/	/	/
6-oxoLCA	18.93 ± 16.57	/	/	/
7-oxoLCA	/	/	/	/
12-oxoLCA	/	/	/	/
6,7-dioxoLCA	ND	/	/	/
7,12-dioxoLCA	4.52 ± 6.37	/	/	/
LCA-3-S	174.03 ± 282.05	7.76 ± 9.24	/	/
GLCA-3-S	7.71 ± 10.4	1.26 ± 1.24	/	/
TLCA-3-S	4.16 ± 4.97	0.77 ± 0.63	/	/
Refs.	[46]	[47]	[50]	[51]

Note: The concentration is expressed in nmol/g, with unit conversion provided in reference [51]. 3-oxoLCA is equivalent to 3-ketoLCA and DehydroLCA; ND: not detected; Symbol “/” indicates not available in the literature.

## Data Availability

No new data were created or analyzed in this study. Data sharing is not applicable to this article.

## Abbreviations

BA, bile acid; BAs, Bile acids; bai, manipulator; BSH, bile salt hydrolases; CA, cholic acid; CAR, constitutive androstane receptor; CDCA, chenodeoxycholic acid; CYP7A1, cholesterol 7α-hydroxylase; CYP27A1, sterol 27 hydroxylase; DON, deoxynivalenol; FABP6, Fatty Acid Binding Protein 6; FXR, farnesoid X receptor; HCC, hepatocellular carcinoma; IBAT, intestinal BAs transporter; IBD, inflammatory bowel disease; IPI-2I, intestinal epithelial cells; LCA, lithocholic acid; LXR, liver X receptor; MASLD, metabolic dysfunction-associated steatotic liver disease; NAFLD, nonalcoholic fatty liver disease; OSTα, organic solute transporter α; OSTβ, organic solute transporter β; PXR, pregnane X receptor; SULT, sulfotransferase; TGF-β, transforming growth factor β; TGR5, G protein-coupled receptor 5; VDR, vitamin D receptor; 5AR, 5α-reductase; 5BR, 5β-reductase; 3α-HSDH, 3α-hydroxysteroid dehydrogenase; 3β-HSDH, 3β-hydroxysteroid dehydrogenase; 7α-HSDH, 7α-hydroxysteroid dehydrogenase; 7β-HSDH, 7β-hydroxysteroid dehydrogenase; 12α-HSDH, 12α-hydroxysteroid dehydrogenase.
